# Impact of Colored Light on Cardiorespiratory Coordination

**DOI:** 10.1155/2013/810876

**Published:** 2013-12-31

**Authors:** Friedrich Edelhäuser, Florian Hak, Ullrich Kleinrath, Birgit Lühr, Peter F. Matthiessen, Johannes Weinzirl, Dirk Cysarz

**Affiliations:** ^1^Integrated Curriculum for Anthroposophic Medicine, University of Witten/Herdecke, 58313 Herdecke, Germany; ^2^Chair for Theory of Medicine, Integrative and Anthroposophic Medicine, University of Witten/Herdecke, 58313 Herdecke, Germany; ^3^Institute for Integrative Medicine, University of Witten/Herdecke, 58313 Herdecke, Germany; ^4^Gemeinschaftskrankenhaus Herdecke, 58313 Herdecke, Germany

## Abstract

*Background*. Light exposure to the eye can influence different physiological functions, for example, the suprachiasmatic nucleus (SCN). By affecting the autonomic nervous system, the SCN may influence the heart rate variability (HRV). Standardized colored light exposure alters HRV but the results are inconsistent. In this study we investigated the effects of nonstandardized red light (approx. 640 nm) and blue (approx. 480 nm) light (approx. 50 lx) on cardiorespiratory coordination and HRV. *Methods*. 17 healthy subjects (7 males, age: 26.5 ± 6.2 years) were exposed to the following sequence (10 minutes each): daylight-red light-daylight-blue light-daylight. Red and blue lights were created by daylight passing through colored glass panes. Spectral measures of HRV (LF: low frequency, HF: high frequency oscillations, and sympathovagal balance LF/HF) and measures of cardiorespiratory coordination (HRR: heart respiration ratio, PCR: phase coordination ratio) were analyzed. *Results*. The LF component increased and the HF component decreased after red light. Consequently, LF/HF increased after red light. Furthermore, during red light HRR and PCR confined to 4 : 1, that is, 4 heartbeats during one respiratory cycle. *Conclusion*. Nonstandardized red and blue lights are able to alter the autonomic control reflected by HRV as well as cardiorespiratory coordination.

## 1. Introduction

During normal vision, light is captured by the rods and cones. This way the circadian clock located in the suprachiasmatic nucleus (SCN) is entrained every day [1, 2]. Hence, light influences many other functions coordinated to the circadian clock. In addition to this impact of the so-called image forming visual system the existence of intrinsically photosensitive retinal ganglion cells (ipRGCs) has been discovered recently [3]. They form a nonimage forming visual system; that is, the light stimulus is not used to form images. Upon light stimulus the ipRGCs show a synaptically driven response (initiated by inputs from rods and cones) and an autonomous response (which is based on the photopigment melanopsin) [4]. The ipRGCs are more sensitive to short-wavelength (blue) light than to long-wavelength (red) light [5]. They also project onto the SCN [6, 7]. Hence, the image forming and the nonimage forming visual system influence the SCN in response to light.

As the SCN has an impact on the autonomic nervous system (ANS) [8, 9] different visual stimuli (e.g., different colors) may alter the ANS differently. Such alterations may be quantified utilizing the analysis of heart rate variability (HRV). In healthy subjects for example, respiration induces a modulation of heart rate mediated by parasympathetic activity (respiratory sinus arrhythmia, RSA) [10]. The extent of such modulations may be analyzed using spectral analysis [11]. In general, high frequency oscillations of heart rate are associated with modulations of the parasympathetic activity and the low frequency oscillations are due to modulations of sympathetic as well as parasympathetic activity. Additional information may be retrieved if cardiorespiratory interaction is analyzed. It has been shown that the oscillations of heartbeat and respiration may intermittently synchronize [12, 13]. During nighttime sleep heart rate and respiration show most likely a 4 : 1-synchronization; that is, 4 heartbeats occur at fixed times within each respiratory cycle [14–17].

Recent attempts to capture alterations of cardiovascular control caused by colored light were heterogeneous. Bright light (≥5000 lx) acutely increased the average heart rate in healthy subjects with blue light being most effective [18]. In neonates with physiologic jaundice the phototherapy increased heart rate and decreased respiratory rate during active sleep compared to active sleep without phototherapy [19]. In healthy subjects visual stimuli with different colors did not change the average heart rate but caused changes of HRV. During exposure to green or red light (700 lx) very low oscillations increased whereas during blue light these oscillations decreased compared to darkness [20]. Another study showed that after exposure to red or blue dim light (illuminance below 1 lux) the low frequency oscillations of HRV increased in healthy subjects with symptoms of anxiety and depression [21]. These studies indicate that each color stimuli may alter cardiovascular regulation differently.

The aim of this study was to investigate simultaneous effects during the exposure to red or blue lights on HRV, respiratory rate, and cardiorespiratory coordination in healthy subjects. We used a “naturalistic setting” which consisted of colored glass panes in front of a window illuminated by daylight. This way the level of illuminance is low (30 to 100 lx) and not standardized.

## 2. Methods

### 2.1. Subjects

20 healthy subjects participated in the study (10 females, average age: 27 ± 6 years). None of the subjects had any history of cardiovascular diseases, especially, nor hypo- or hypertension or antiarrhythmic therapy. None of the subjects had any experience with any kind of light therapy. Three male subjects had to be excluded from the study because they deliberately changed their respiration. Hence, these subjects not only focused on the different visual stimuli but also on respiration. All subjects gave their written informed consent. The study was approved by the ethics committee of the University of Witten/Herdecke.

### 2.2. Protocol

The subjects sat comfortably in an armchair approx. 1.5 m in front of a window. The size of the window was matched to the size of the colored glass panes (approx. 0.6 m × 1.1 m) with black opaque curtains. A red and a blue glass pane were used to generate two different color stimuli. (The red colored pane was produced by addition of gold, the blue colored pane by addition of ferrous oxide. They were manufactured by Lucien Turci and Marianne Altmaier, Lichtblick e.V., Lörrach, Germany.) Each glass pane was mounted on a stand with reels which allowed the quick change of the visual stimuli. During color vision the black opaque curtains were placed such that color light was the only visual stimulus. Each subject was exposed to the following sequence of visual stimuli (level of illuminance: 30 to 100 lx):

(1) $$\begin{array}{c} \text{daylight-red}\;\;\text{ligh-daylight-blue}\;\;\text{light-daylight}. \end{array}$$

The duration of each stimulus was 10 min (total duration: 50 min). In order to minimize a bias caused by circadian variations of HRV [22] the procedure was carried out between 10 am and 1 pm.

### 2.3. Data Acquisition

A 1-channel Holter electrocardiogram (ECG) and a respiratory trace (nasal/oral airflow captured by a thermocouple) were recorded during each session (Medikorder MK2, Schiller Engineering, Graz, Austria). The device's sampling rate of the ECG was 3000 Hz and, hence, the times of the device's internal R-peak detection had a precision <1 ms. To reduce memory consumption, the ECG was saved at a sampling rate of 250 Hz and the nasal airflow was saved at a sampling rate of 100 Hz. After transferring the ECG, the airflow trace and times of the R-peaks to a personal computer, the ECG, and the times of the R-peaks were inspected visually. In case of artefacts the times of associated R-peaks were corrected where necessary (e.g., by removing artifacts or by correcting the time of the R-peak). Less than 0.01% of R-peaks identified by the device had to be corrected.

The airflow trace was analyzed as follows. Inspiratory onsets were defined as local minima of the airflow because they were caused by the change from exhaling warm air (warmed be the respiratory tract) to inhaling air at the temperature of the environment. The times of the automatically identified inspiratory onsets were also inspected visually. Again, in case of artifacts the timings of inspiratory onsets were corrected.

The visual inspection of R-peaks, the subsequent HRV analysis (see Section 2.4) and the analysis of the airflow trace was carried out using custom programs written with Matlab (The Mathworks, Natick, MA, USA).

### 2.4. Heart Rate Variability

The normal-to-normal intervals between successive R-peaks (RR-interval series) served as the basis for the calculations. To avoid effects of transitions between different visual stimuli only the 5-minute epoch in the middle of each visual stimulus was analyzed. The mean normal-to-normal intervals and the accompanying standard deviation (SDNN) were calculated as basic time domain parameters. In the frequency domain, the extent of very low, low and high frequency oscillations of heart rate variations (VLF: ≤0.04 Hz, LF: 0.04–0.15 Hz, and HF: 0.15–0.4 Hz) and the ratio LF/HF were calculated using the fast Fourier transformation [11]. In addition to the spectral measures of HRV we also quantified self-similarity of the heart rate time series using the Detrended Fluctuation Analysis (DFA) [23, 24].

### 2.5. Cardiorespiratory Coordination

The mean respiratory rate and its standard deviation were calculated. Cardiorespiratory coordination was quantified by two different approaches. (a) The ratio of heart rate and respiratory rate (HRR, heart respiration ratio) served as a simple indicator of intermittent cardiorespiratory coordination [25]. (b) Furthermore, the “phase coordination ratio” (PCR) [14, 26] was calculated because it also takes into account the temporal coordination of heartbeat and respiration in more detail. This approach is advantageous compared to for example, analyzing cardiorespiratory synchronization using so-called synchrograms [12] because it is able to detect short and intermittent epochs of cardiorespiratory coordination (coordination can be detected after only two consecutive respiratory cycles) [14], whereas the analysis of synchrograms needs longer epochs to detect cardiorespiratory synchronization [13, 17].

The analysis was carried out as follows. First, the RR-tachogram RR_*i*_ (*i* = 1,…, *N*) was transformed into a symbolic sequence symbolizing the acceleration and deceleration of heart rate (Figure 2):

(2) $$\begin{array}{c} S_{i}=\{\begin{array}{cc} 0, & \text{if}\;\;\text{R}{\text{R}}_{i}-\text{R}{\text{R}}_{i-1}\geq 0,\\ 1, & \text{if}\;\;\text{R}{\text{R}}_{i}-\text{R}{\text{R}}_{i-1}<0. \end{array} \end{array}$$

**Figure 1:**
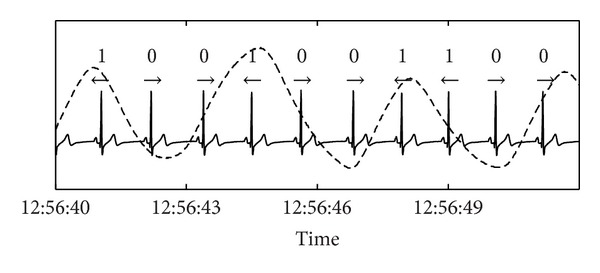
A short example of an ECG recording and the respiratory trace (dashed line). The symbols denote the encoding of acceleration (1) and deceleration (0) of the instantaneous heart rate as a consequence of respiratory sinus arrhythmia, that is, the modulation of heart rate by respiration. The sequence of binary symbols can be used to detect cardiorespiratory coordination. In this example, a cardiorespiratory coordination with a ratio of 7 : 2 (7 heartbeats in 2 respiratory cycles) would be detected.

Next, *m* : *n*-coordination of heartbeat and respiration leads to specific sequences of 0 s and 1 s. For example, a 7 : 2-coordination (7 heartbeats during 2 respiratory cycles) is unambiguously characterized by the following binary pattern (Figure 1): 1001100. To minimize spurious detection of cardiorespiratory coordination the respective pattern must appear and also at least three subsequent symbols must belong to the same pattern. The following 7 *m* : *n*-ratios of cardiorespiratory coordination contain large parts of relevant information: 3 : 1, 7 : 2, 4 : 1, 9 : 2, 5 : 1, 11 : 2, and 6 : 1 [14]. The binary patterns reflecting these *m* : *n*-ratios are listed in Table 1. Subsequently, the PCR was calculated as the weighted mean of the detected *m* : *n*-ratios:

(3) $$\begin{array}{c} \text{PCR}=\frac{\sum_{i=1}^{7}m:n\cdot N\left(m:n\right)}{\sum_{i=1}^{7}N\left(m:n\right)}. \end{array}$$

Here, *N*(*m* : *n*) is the number of occurrences of the respective *m* : *n*-ratio. To quantify cardiorespiratory coordination HRR and PCR were calculated for the middle 5-minute epochs of each visual stimulus.

### 2.6. Statistics

The objective of this exploratory pilot study was to assess the effects of red and blue visual stimuli on HRV and cardiorespiratory coordination. Each 5-minute epoch was quantified by the following parameters: mean RR-interval, SDNN, VLF, LF, HF, LF/HF, mean and standard deviation of respiratory rate, HRR, and PCR. To minimize effects of transitions between successive visual stimuli the 5-minute epoch in the middle of each visual stimulus was analysed. A nonparametric 1-way analysis of variance with repeated measurements (Friedman test) was used to quantify differences within the succession of different visual stimuli. Post hoc differences between different visual stimuli were calculated using mean ranks and a correction for multiple comparisons according to Bonferroni. A *P* < 0.05 was considered statistically significant.

A specific feature of HRR is the centering towards a ratio of 4 : 1 during nighttime sleep [17, 27]. We analyzed whether cardiorespiratory coordination centers more strict to this 4 : 1-ratio during the exposure to colored light. Centering of HRR towards 4 : 1 during color light is defined as

(4) $$\begin{array}{c} \text{HR}{\text{R}}_{\text{dur}\_\text{col}}-\text{HR}{\text{R}}_{\text{bef}\_\text{col}}>0,\;\text{if}\;\text{HR}{\text{R}}_{\text{bef}\_\text{col}}<4:1,\\ \text{HR}{\text{R}}_{\text{dur}\_\text{col}}-\text{HR}{\text{R}}_{\text{bef}\_\text{col}}<0,\;\text{if}\;\text{HR}{\text{R}}_{\text{bef}\_\text{col}}>4:1. \end{array}$$

Furthermore, the larger the deviation of HRR before the color stimulus (HRR_bef_col_) from the 4 : 1-ratio the larger the corresponding difference to achieve a centring (or confinement) towards 4 : 1 during the color stimulus (HRR_dur_col_). To quantify this centring, we plotted HRR_bef_col_ versus (HRR_dur_col_ − HRR_bef_col_). According to the definition, centring is indicated by a negative correlation in this diagram. Hence, we calculated Pearson's correlation coefficient *r* and its accompanying *P* value. Spurious correlations caused by outliers were detected as follows: the correlation coefficient was also calculated omitting the points of minimal and maximal difference HRR_dur_col_ − HRR_bef_col_. The correlation was considered significant only if the *P*-value of both correlations (original and without outliers) was *P* < 0.05. The centering of PCR towards the 4 : 1-ratio was analyzed analogously.

## 3. Results

**Figure 2:**
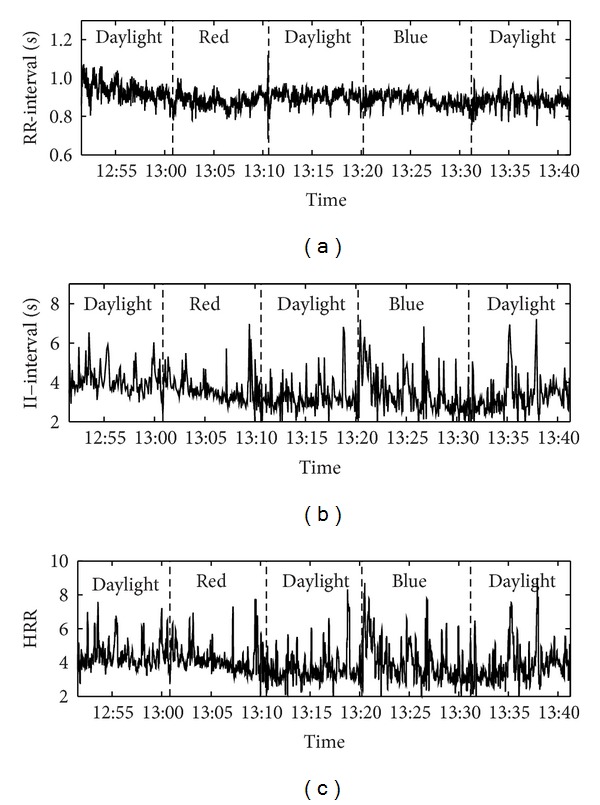
Example of the RR-interval series, II-interval series, and the heart respiration ratio (HRR) of one subject. The sequence of the visual stimuli is shown in each diagram.

An example of a complete RR-interval series, II-interval series, and heart respiration ratio (HRR) is shown in Figure 2. Note that the 5-minute epoch in the middle of each visual stimulus was analyzed. The level of illuminance was approx. 50 lx during colored light. The average RR-interval was 1002 ms (i.e., approx. 60 beats per minute) at the beginning and did not change during the succession of the different visual stimuli (see Table 2). SDNN as a measure in the time domain was constant throughout the procedure (73 ms). In the frequency domain LF, HF, and LF/HF showed variations with respect to the sequence of the visual stimuli, whereas the changes of VLF were slightly above the level of significance (*P* = 0.0526). LF was lower during daylight at the beginning compared to daylight in the middle (6.84 ln ms^2^ versus 7.31 ln ms^2^, *P* < 0.05) and daylight at the end (6.84 ln ms^2^ versus 7.39 ln ms^2^, *P* < 0.05). Furthermore, LF was also lower during red light compared to daylight at the end (6.94 ln ms^2^ versus 7.39 ln ms^2^, *P* < 0.05). On the contrary, HF was only higher during daylight at the beginning compared to daylight in the middle (7.33 ln ms^2^ versus 7.02 ln ms^2^, *P* < 0.05). LF/HF was lower during daylight at the beginning compared to daylight in the middle (−0.24 versus 0.47; *P* < 0.01) and compared to daylight at the end (−0.23 versus 0.64, *P* < 0.01). With respect to fractal properties of heart rate variations the self-similarity parameter *α* was also lower during daylight at the beginning compared to daylight in the middle, blue light and daylight at the end (0.80 versus 1.03, *P* < 0.01; 0.80 versus 1.06, *P* < 0.05 and 0.80 versus 1.08, *P* < 0.01).

The average respiratory rate was 5.5 to 16.4 cycles per minute (cpm). The standard deviation of respiratory rate ranged from 2.3 to 3.0 cpm. Both parameters did not change during the succession of the different visual stimuli. The average  : *n*-ratio of cardiorespiratory coordination as quantified by HRR and PCR did not also change. They were both close to 4 during the different visual stimuli.

**Figure 3:**
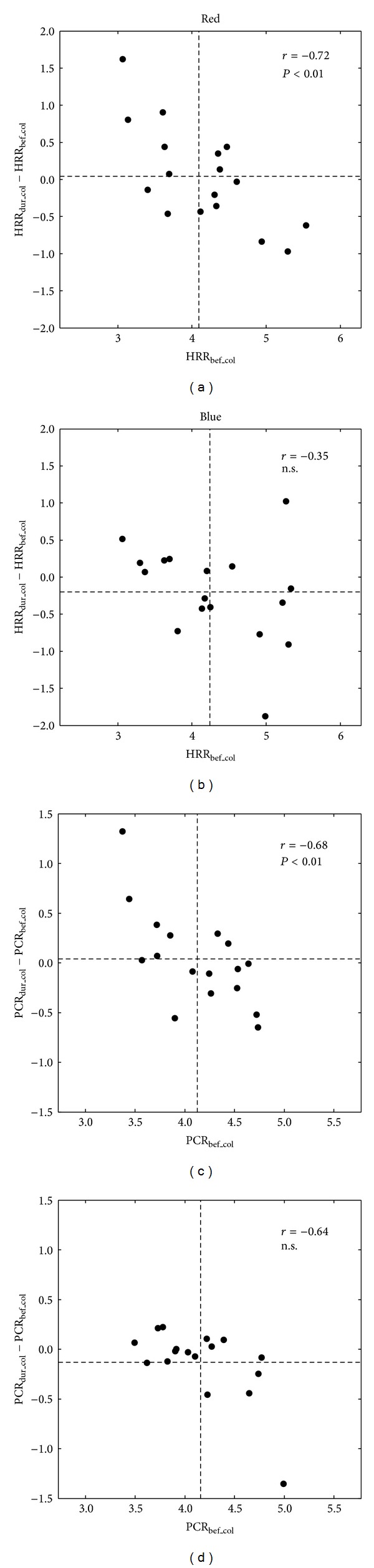
Analysis of centering of cardiorespiratory coordination towards 4 : 1 during exposure to red and blue color lights. (a), (c) During red light the significant negative correlation coefficient *r* for HRR and PCR indicates a centering towards 4 : 1. (b), (d) During blue light a centring does not occur; that is, the correlation is not significant. Note that in diagram (d) the high correlation *r* is spuriously caused by the outlier in the lower right part of the diagram. Dashed lines indicate average HRR_bef_col_ (and PCR_bef_col_) and average HRR_dur_col_ − HRR_bef_col_ (and PCR_dur_col_ − PCR_bef_col_), respectively.

The centring (or confinement) of cardiorespiratory coordination with respect to the 4 : 1-ratio was as follows. During red light a clear linear relation was found in the centering diagram as indicated by the correlation coefficient *r* (see Figures 3(a) and 3(c); HRR: *r* = −0.72, *P* < 0.01; PCR: *r* = −0.68, *P* < 0.01). Hence, the larger the deviation from 4 during daylight before the color stimulus the larger the difference HRR_dur_col_ − HRR_bef_col_ to achieve the centring (confinement) during the color light stimulus. As centring leads to a narrower distribution of HRR (and PCR) the standard deviation of HRR (and PCR) was also lower during red light (see Table 1). Note that centring does not affect the average HRR (and PCR). During blue light HRR and PCR did not centre (Figures 3(b) and 3(d); HRR: *r* = −0.35, n.s., PCR: *r* = −0.64, n.s.). Note that the relatively large correlation coefficient in Figure 3(d) is caused by the outlier (correlation coefficient without outlier: *r* = −0.44, *P* = 0.10). We also checked centring diagrams for all other combinations of visual stimuli. None of these centring diagrams showed a significant negative correlation, that is, centring towards the 4 : 1-ratio.

## 4. Discussion

Visual stimuli of red and blue light without standardization of illuminance did not change average heart rate and respiratory rate throughout the procedure. In brief, LF oscillations increased during the procedure whereas HF oscillations decreased during the first part of the procedure. LF/HF showed more detailed results: this parameter only increased during the exposure to daylight. That is, red and blue lights did not alter LF/HF, whereas daylight in the middle and daylight at the end did. The self-similarity parameter *α* increased during daylight at the end compared to daylight at the beginning. Furthermore, during red light cardiorespiratory coordination as expressed by HRR and PCR centred (confined) towards 4 : 1 (4 heartbeats in one respiratory cycle). That is, HRR and PCR were closer to 4 : 1 during red light than during daylight at the beginning. Blue light did not show any effect on cardiorespiratory coordination.

Recent studies showed clear effects of visual stimuli on heart rate and HRV. Healthy subjects exposed to bright light (>5000 lx) showed an increase of the average heart rate [18]. The authors state that the increase of heart rate may be primarily dependent on the light-induced increase of sympathetic activity of the ANS. In another study, HRV was altered during the exposure to colored light for 10 minutes, whereas the average heart rate did not change [20]. VLF oscillations increased during red and green lights, whereas they decreased during blue light. Straight after the color stimuli HRV returned to the values that were observed before the color stimulus. The origins of the VLF component are still a matter of debate [11, 28]. Some authors suggest that both, the very low frequency variations of vagal baroreflex sensitivity and the very low frequency variations of HRV, are caused by vagal modulations [29]. Hence, the changes of the VLF component could be cautiously interpreted in terms of changes of vagal activity. LF oscillations did not change and HF oscillations decreased during green light only. Hence, vagal activity decreased during green light. These results were obtained only during the visual stimulation with a standardized level of illuminance (700 lx). Visual stimuli with dim light (1 lx) also led to alterations of the ANS [21]. However, these results were obtained after the visual stimulation. Heart rate decreased after visual stimulation with dim red, blue, and green lights compared to baseline. LF oscillations increased after red and blue lights, whereas HF decreased after red light. As a consequence, the sympathovagal balance LF/HF decreased after red light.

In the present study we found an increase of LF oscillations and a decrease of HF oscillations after the visual stimulation with red light. That is, sympathetic oscillations increased, whereas parasympathetic oscillations decreased after red light. Consequently, LF/HF increased. These findings are in accordance with the above mentioned findings of Choi et al. [21]. Furthermore, we found an increase of the self-similarity parameter *α* after the red light stimulus. This increase is associated with changes in the overall spectral density as *α* reflects the 1/*f* ^*β*^ decay of the spectrum. In this particular case, more power is shifted to lower frequencies after the red light stimulus. In contrast to previous findings, we did not find any alteration of the average RR-interval, that is, average heart rate, caused by a color light stimulus. The differences during the color light stimulus as described by Schäfer and Kratky [20] could also not be confirmed.

The differences of the results with respect to the study by Schäfer and Kratky could be caused by the different levels of illuminance (700 lx versus 30 to 100 lx). Even low levels of illuminance may have an impact on the ANS because the ipRCGs of the nonimage forming visual system even respond to few photons [30] and, hence, may also affect the SCN and the ANS. The synaptically mediated response of the ipRCGs caused by low light stimulus [4] supports this notion.

The only simultaneous effect during colored light exposure was the centring of cardiorespiratory coordination towards 4 : 1 during red light as indicated by HRR and PCR. Note that HRR is a simple measure of cardiorespiratory coordination [25], whereas PCR is more strict with respect to the temporal coordination of heart beat and respiration [14]. Hence, during red light the coordination was adjusted such that the 4 : 1-ratio emerged. Such a coordination has been observed in healthy subjects during quiet rest [12, 13] as well as during nighttime sleep [17, 27, 31]. The emergence of this coordination indicates a process of relaxation and recovery of the organism. We note that this kind of relaxation is different from, for example, an increase of the high frequency oscillations of HRV as an indicator of increased vagal activity [32].

With respect to the limitations we note that especially the effects of blue light may be confounded by the previous red light stimulus. The response of the ipRGCs upon visual stimuli is more sustained than that of conventional RGC [4]. Hence, carry over effects could be responsible for the results of the blue light stimulus. A randomized balanced design with respect to the succession of colored light stimuli may also reveal specific effects of blue light on HRV or cardiorespiratory coordination. Ideally, each color stimulus should be investigated separately to avoid carry over effects. The increase of LF oscillations and decrease of HF oscillations during the procedure could be avoided if more time would be allowed to acclimatize before starting the procedure. However, a longer adaptation period could also lead to an increased tiredness caused by low levels of alertness during the adaptation period.

In conclusion, the exposure to red light showed a centering of cardiorespiratory coordination towards 4 : 1. Furthermore, red and blue lights altered autonomic nervous functions as expressed by HRV. These results may lead to a rationale for the application of colored light as a therapeutic means as used in Anthroposophic Arts Therapies. The physiological mechanisms leading to these alterations need to be explored.

## Figures and Tables

**Table 1:** Binary patterns used for the analysis of the different *m* : *n*-ratios of cardiorespiratory coordination.

*m* : *n*-ratio	Binary pattern	Complementary binary pattern
3 : 1	001001	110110
7 : 2	0010011	1101100
4 : 1	00110011	11001100
9 : 2	000110011	111001100
5 : 1	0001100011	1110011100
11 : 2	00011000111	11100111000
6 : 1	000111000111	111000111000

**Table 2:** Results of HRV and cardiorespiratory coordination. All values are mean ± SD.

	Daylight	Red light	Daylight	Blue light	Daylight
RR-interval (ms)	1002 ± 149	999 ± 132	992 ± 123	995 ± 1113	986 ± 108
SDNN (ms)	73 ± 31	74 ± 33	79 ± 35	78 ± 33	83 ± 28
VLF (ln ms^2^)	6.61 ± 0.75	6.88 ± 0.75	6.99 ± 0.83	7.31 ± 0.75	7.51 ± 0.75
LF (ln ms^2^)^§§^	6.84^∗,#^ ± 0.88	6.94* ± 0.98	7.31 ± 1.07	7.18 ± 0.99	7.39 ± 0.77
HF (ln ms^2^)^§^	7.33^#^ ± 1.00	7.10 ± 1.00	7.02 ± 1.03	6.91 ± 1.23	6.86 ± 1.06
ln (LF/HF)^§§§^	−0.24^∗∗,##^ ± 0.93	0.12 ± 0.87	0.47 ± 0.91	0.39 ± 0.74	0.64 ± 0.81
*α* ^§§^	0.80^∗∗,##,$^ ± 0.19	0.94 ± 0.22	1.03 ± 0.23	1.06 ± 0.24	1.08 ± 0.19
Resp. rate (min^−1^)	15.5 ± 2.0	15.6 ± 2.0	15.8 ± 2.0	16.4 ± 2.1	16.2 ± 2.8
SD (resp. rate) (min^−1^)	2.3 ± 0.9	2.4 ± 1.0	3.0 ± 0.9	3.0 ± 1.2	2.9 ± 1.8
HRR	4.1 ± 1.2	4.2 ± 1.1	4.2 ± 1.2	4.0 ± 1.2	4.2 ± 1.3
PCR	4.1 ± 1.1	4.2 ± 1.1	4.1 ± 1.2	4.0 ± 1.1	4.0 ± 1.1

^§^ *P* < 0.05, ^§§^ *P* < 0.01, and ^§§§^ *P* < 0.001; *P < 0.05 versus daylight (end); **P < 0.01 versus daylight (end); ^#^ *P* < 0.05 versus daylight (middle); ^##^ *P* < 0.01 versus daylight (middle); ^$^ *P* < 0.05 versus blue light.
